# Effects of the Glycosylation of the HA Protein of H9N2 Subtype Avian Influenza Virus on the Pathogenicity in Mice and Antigenicity

**DOI:** 10.1155/2024/6641285

**Published:** 2024-05-17

**Authors:** Bing Liang, Menglu Fan, Qi Meng, Yaping Zhang, Jiayu Jin, Na Chen, Yuanlu Lu, Chenfeng Jiang, Xingxing Zhang, Zongyou Zou, Jihui Ping, Juan Su

**Affiliations:** ^1^MOE International Joint Collaborative Research Laboratory for Animal Health and Food Safety and Jiangsu Engineering Laboratory of Animal Immunology, College of Veterinary Medicine, Nanjing Agricultural University, Nanjing, 210095, China; ^2^State Key Laboratory of Veterinary Biotechnology, Harbin Veterinary Research Institute in CAAS, Harbin, China

## Abstract

As the H9N2 subtype avian influenza virus (H9N2 AIV) evolves naturally, mutations in the hemagglutinin (HA) protein still occur, which involves some sites with glycosylations. It is widely established that glycosylation of the H9N2 AIV HA protein has a major impact on the antigenicity and pathogenicity of the virus. However, the biological implications of a particular glycosylation modification site (GMS) have not been well investigated. In this study, we generated viruses with different GMSs based on wild-type (WT) viruses. Antigenicity studies revealed that the presence of viruses with a 200G^+^/295G^−^ mutation (with glycosylation at position 200 and deletion of glycosylation at position 295 in the HA protein) combined with a single GMS, such as 87G^+^, 127G^+^, 148G^+^, 178G^+^, or 265G^+^, could significantly affect the antigenicity of the virus. Pathogenicity assays revealed that the addition of GMS, such as 127G^+^, 188G^+^, 148G^+^, 178G^+^, or 54G^+^, decreased the virulence of the virus in mice, except for 87G^+^. The removal of GMS, such as 280G^−^ or 295G^−^, increased the pathogenicity of the virus in mice. Further studies on pathogenicity revealed that 87G^+^/295G^−^ could also enhance the pathogenicity of the virus. Finally, we selected the WT, WT-87G^+^, WT-295G^−^, and WT-87G^+^/295G^−^ strains as our further research targets to investigate the detailed biological properties of the viruses. GMS, which can enhance viral pathogenicity, did not significantly affect replication or viral stability *in vitro* but significantly promoted the expression of proinflammatory factors to enhance inflammatory responses in mouse lungs. These findings further deepen our understanding of the influence of the glycosylation of the HA protein of H9N2 AIV on the pathogenicity and antigenicity of the virus in mice.

## 1. Introduction

SARS-CoV-2 has recently become common, and there have been cases of coinfection with influenza viruses that have resulted in more serious clinical symptoms, posing a significant threat to public health [1]. Moreover, the damage caused by H9N2 AIV is far-reaching [2]. First, poultry infected with H9N2 AIV will display clinical symptoms such as egg reduction. Typically, a substantial amount of poultry is kept in large and intensive environments on farms, and due to the high transmissibility of low-pathogenicity avian influenza virus, the economic impact of an outbreak of AIV can be incredibly damaging [3–5]. In addition, chickens infected with AIV are more likely to be coinfected with other pathogens due to the low resistance of the immune system, resulting in a high fatality rate [6, 7]. People with high-risk factors, such as farm breeders who come into contact with poultry and those who work in live poultry markets, have an increased likelihood of being infected with AIVs with low pathogenicity. In these populations, H9N2 AIV is often detected in the serum, yet infected individuals usually do not exhibit any clinical signs [4, 8–11]. In 2013, according to the gene sequences of H7N9 and H10N8 strains isolated from humans, six genes were identified from H9N2 AIV. Therefore, H9N2 AIV also acts as a genetic contributor [12–15]. Vaccines have been used to immunize chickens in China since 1998. However, with the continuous antigenic evolution of H9N2 AIV, it has been difficult for vaccines to provide comprehensive protection for chickens [16, 17]. H9N2 AIV has become the most widespread subtype of influenza virus in China and has seriously affected the development of the poultry industry [18]. H9N2 AIV is continuously mutating under chronic immune stress. One of the key factors that can evade vaccine immunity is the HA gene [19]. These phenomena are closely related to the glycosylation of the H9N2 AIV HA protein, as the deletion or addition of GMS to the HA protein affects antigenicity, pathogenicity, and even receptor binding [20–22]. Collectively, these findings suggest that we must be mindful of the potential interspecies transmission ability of H9N2 AIV and investigate the changes in antigenicity and pathogenicity caused by the glycosylation of the H9N2 AIV HA protein during the evolution of the virus [23, 24].

Glycosylation of proteins has gradually been recognized as an important process during viral evolution [25]. AIV produces two membrane-bound surface glycoproteins, hemagglutinin (HA) and neuraminidase (NA), which include N-linked oligosaccharides. HA is a trimer, and its monomer consists of the head (HA1) and stem (HA2) regions. N-glycosylation is a kind of posttranslational modification of mammalian glycoproteins that involves the attachment of oligosaccharides to Asn residues of the Asn-X-Ser/Thr-Y motif, where X/Y can be any amino acid other than proline [26]. The number of GMSs on the HA protein of the human seasonal influenza virus has risen with time [27]. Identifying potential glycosylation sites is an effective strategy for preventing host immunological pressure on influenza viruses because the glycosylation of HA normally influences the antigenicity and pathogenicity of the virus [28].

Glycosylation of the HA protein blocks antibodies from binding, thus protecting against viruses [29]. The GMS on the HA globular head has been revealed to be particularly important for the antigenicity and receptor-binding characteristics of influenza virus [22, 30]. GMS differences in H3N2 subtype influenza viruses impact the antibody neutralization response to influenza vaccine strains, decreasing the efficacy of seasonal influenza vaccines [31]. The insertion of a novel N-linked oligosaccharide on the HA of H3N2 subtype influenza viruses resulted in immunological escape from antibody pressure by modifying their antigenicity [32].

The glycosylation of the influenza virus HA protein is an essential factor in viral pathogenicity. The introduction of glycosylation at site 158 (H9: position 152) of the H5N1 subtype influenza virus can promote viral production and intensify the host response, thus increasing its pathogenicity in mice [33]. The presence of N127D on the HA of H9N2 AIV suggested that the glycosylation at 127 was eliminated, which decreased the pathogenicity of the virus in mice [22]. The HA glycosylation of H3N2 subtype influenza viruses has been shown to increase annually, and the increase in GMS has resulted in a decrease in its pathogenicity in mice. The more glycosylated modifications there are on the virus surface, the more sensitive the virus is to lectin in the host body, and it is easier for lectin to neutralize and inhibit the influenza virus [34].

Distinct GMSs can be found in various strains of different subtypes of influenza virus [35]. Since the GMS of HA has varying effects on viruses and there are different GMSs on the HA protein that exist naturally, only a few sites have been identified in existing reports and still need to be better understood. Thus, it is highly important to investigate the biological significance of these GMSs comprehensively. In this study, the HA gene from the A/chicken/Anhui/99/2017 strain was utilized to construct the pHH21-99-HA plasmid as a site-specific mutation template to rescue these different GMS viruses. Then, we explored the effects of different GMSs on the HA of H9N2 AIV on the antigenicity and pathogenicity of the virus and screened out the functional GMS.

## 2. Materials and Methods

### 2.1. Ethics Statement

All experimental animals used in this investigation were housed in labs with level 2 biosafety. Mice were treated humanely in accordance with the People's Republic of China's Animal Ethics Regulations and Guidelines as well as the regulations of Nanjing Agricultural University's Animal Protection and Use Committee (SYXK(Su)2021-0086).

### 2.2. Cells and Viruses

This study used Madin–Darby canine kidney (MDCK) cells, human embryonic kidney cells (293T), human lung adenocarcinoma epithelial (A549) cells, and Vero cells. The virus titers in MDCK cells were measured using a plaque assay. The medium used was high-glucose DMEM (Gibco) supplemented with 10% FBS, and the cells were incubated at 37°C and 5% CO_2_. This study used a 12-plasmid reverse genetic technique to create recombinant viruses, with the internal genes derived from A/chicken/Jiangsu/875/2018 and the HA and NA genes from A/chicken/Anhui/99/2017 [36].

### 2.3. Construction of a Three-Dimensional Model of HA Protein

The amino acid sequence of the A/chicken/Anhui/LH99/2017 strain was added to the SWISS-MODEL website (https://swissmodel.expasy.org/interactive) to generate a trimeric model of the HA protein, and this model was subsequently imported into PyMOL software (DeLano Scientific LLC) for simulation. Different glycosylation sites and RBS regions are marked with the indicated colors.

### 2.4. Generation of GMS HA Plasmids

Using pHH21-99-HA as the template, all plasmids with different GMSs were constructed by site-specific mutation, and Oligo 7.0 software was used to design specific primers. The detailed primer information is provided in Table S3 in Supplementary Materials. The following materials were added to 0.2 ml PCR tubes: dNTP mix (10 mM). Next, 1 *μ*l of Phanta® Max Buffer, 25 *μ*l of Phanta® Max Super-Fidelity DNA Polymerase, 50 *μ*l of ddH_2_O, 500 ng of plasmid template, and 1 *μ*l each of upstream and downstream mutant primers were added. The PCR conditions were as follows: 5 min of predenaturation at 95°C, 30 s of denaturing at 95°C, 30 s of annealing at 58°C, and 25 cycles of extension at 72°C (1,000 bp/1 min). After an additional 10 min at 72°C, the temperature was maintained at 16°C. The following reaction system was used to digest the methylated plasmid template using Dpn I restriction endonuclease: 10x buffer (5 *μ*l), Dpn I enzyme (1 *μ*l), and 50 *μ*l of PCR products. Reaction conditions: 30 min in a water bath at 37°C. Subsequently, the digested products were transformed into *E. coli* DH5*α*, and then, positive monoclonal colonies were selected. Positive plasmid screening and sequencing were used to verify the success of the point mutation.

### 2.5. Virus Rescue

Several GMS viruses were rescued using the 12-plasmid reverse genetic method [36]. pCAGGS-WSN-PB2, pCAGGS-WSN-PB1, pCAGGS-WSN-PA, and pCAGGS-WSN-NP (1 *μ*g each) were used. pHH21-99-HA (various GMS HAs), pHH21-99-NA, pHH21-875-PB2, pHH21-875-PB1, pHH21-875-PA, pHH21-875-NP, pHH21-875-M, and pHH21-875-NS (0.2 *μ*g each) were used. The plasmids were transfected into 293T cells at an 80% confluence using Lipo2000 (2 *μ*l per time, Invitrogen), and the cells were subsequently grown in dishes with a diameter of 35 mm. TPCK trypsin was added 24 hr after transfection, and the cell suspension was inoculated into SPF chicken embryos aged 9–11 days at 48 hr after transfection and incubated in a 37°C incubator for 48 hr for virus proliferation. The allantoic fluid of the chicken embryo was collected, and then, the HA titers were determined. The virus titer was calculated by the number of plaques it formed on MDCK cells and was expressed as PFU/ml.

### 2.6. Antigenic Analysis

A total of 30 GMS viruses, WT, R-1–R-29 (different GMS viruses with various glycosylation sites are shown in Table 1) virus allantoic fluid, were added to *β*-propiolactone (at a 1 : 2,000 vol:vol ratio) to inactivate the virus in this way. Then, the inactivated viruses were made into oil emulsion-inactivated vaccines by adding Tween 80 and Span, and SPF chickens aged 4–6 weeks were immunized with multiple subcutaneous and intramuscular injections. At 21 days after immunization, blood samples were collected, and the serum was separated. Cross-hemagglutination inhibition experiments were subsequently conducted. An antigen map was drawn according to the hemagglutination inhibition results (https://acmacs-web.antigenic-cartography.org/).

### 2.7. Mouse Experiments

The Xipuer-Bikai Experimental Animal Company supplied pathogen-free 4-week-old female BALB/c mice (Shanghai, China). Eight mice per group were intranasally inoculated with a volume of 50 *μ*l containing 4 × 10^5^ PFU of various glycosylated recombination viruses, and their body weights were monitored until the 14th day postinfection. Mice in the negative control group received 50 *μ*l of PBS solution intranasally. On the third day after infection, lung samples were taken from three mice and used to titrate the virus. The mice were euthanized when their weights were less than 75% of their initial body weights. The median lethal dose test (MLD50) for GMS viruses in mice: Three mice were infected with recombinant viruses at doses of 10^4^, 10^5^, and 10^6^ PFU in a 50 *μ*l volume. The mice were fed for 14 days, after which their daily weight changes and mortality rates were recorded. The MLD50 was estimated using Reed and Muench's approach.

### 2.8. Western Blotting

To extract the total protein from the virus, a single layer of MDCK cells at a density of 95% was infected with the recombinant virus at an MOI of 1. The cells were then lysed with NP40 18–24 hr after infection. After being mixed with 4x loading buffer (Solarbio, Beijing), the treated cells were heated to 100°C for 15 min to denature them. Following separation via 10% polyacrylamide-containing SDS‒PAGE with 10 *μ*l of each extract, the proteins were transferred to an acetate membrane (GE Healthcare, Amersham), which was subsequently blocked with 5% skim milk and treated overnight at 4°C with a 1 : 1,000 laboratory-made anti-H9N2 HA monoclonal antibody. The membrane was washed five times before being incubated for 1 hr at 37°C with a sheep antimouse secondary antibody conjugated to HRP (1 : 10,000; KPL, Gaithersburg, MD). After that, the membrane was washed again, and enhanced chemiluminescence (Vazyme, Nanjing) was used to detect the target protein bands.

### 2.9. Receptor-Binding Assay

Two glycopolymers were utilized in solid-phase direct binding studies to assess the receptor-binding properties of the virus. They were *α*-2,3-sialylglycopolymer [Neu5Ac*α*2-3Galb1-4GlcNAcb1-pAP (para-aminophenyl)-*α*-polyglutamic acid (*α*-PGA)]. The molecule Neu5Ac*α*2-6Galb1-4GlcNAcb1-pAP (para-aminophenyl)-alpha-polyglutamic acid (*α*-PGA) is also present [37, 38]. In this investigation, a goat anti-chicken antibody (Sigma‒Aldrich, St. Louis, MO, USA) and horseradish peroxidase (HRP) were used for ELISAs with chicken serum generated from an inactivated virus. A microplate reader was used to measure the absorbance at 490 nm. The dose–response curves of virus binding to glycopolymers were analyzed using a single-site binding algorithm and curve fitting with GraphPad Prism to obtain the constant association values (Ka). The values displayed are the average ± standard deviation of three individual tests, each carried out in triplicate.

### 2.10. Real-Time RT-PCR

To detect four cytokines, namely, IL-1*β*, IL-6, TNF-*α*, and IFN-*β*, the ID numbers of each cytokine were obtained from the National Center for Biotechnology Information (NCBI) database and then input into GenBank, with house mice as the species used for primer retrieval. The top cytokine primer sequences were selected and are shown in Table S4 in Supplementary Materials. One milliliter of PBS was added to the mouse lung sample, the mixture was ground, and the supernatant was obtained by centrifugation at 12,000 rpm for 10 min at 4°C. The TRIzol method was used to extract total RNA, which was subsequently reverse-transcribed into cDNA (Vazyme, Nanjing). Using a Light Cycler® 96 system, qRT-PCR was carried out using a 20 *μ*l system, 2 *μ*l of cDNA, and 1 *μ*l of each of the downstream and upstream primers. Briefly, 10 *μ*l of 2x AceQ qPCR Probe Master Mix and 6 *μ*l of ddH_2_O were added, followed by 40 cycles of 95°C for 5 min, 95°C for 10 s, and 60°C for 30 s. The relative expression of mRNA was standardized to the aldehyde-3-phosphate dehydrogenase (GAPDH) level using the 2^−*ΔΔ*Ct^ method.

### 2.11. Viral Growth Kinetics Analysis

Monolayered MDCK cells and A549 cells were inoculated with WT, WT-87G^+^, WT-295G^−^, or WT-87G^+^/295G^−^ at MOIs of 0.01 and 0.5, respectively. The cell supernatants were collected at 12, 24, 48, and 60 hr after inoculation, and plaque tests were used to measure the virus titer and plot the virus growth curve on several cell lines.

### 2.12. Thermal Stability Assay

The HA titer of several recombinant viruses was determined by incubating them in a water bath at 56°C for 0, 5, 10, 15, 30, 60, 90, 120, and 150 min. The thermal stability curves of different recombinant viruses were constructed according to the HA titer losses.

### 2.13. pH Stability

Equal amounts of 100 mM phosphate buffer (pH = 6.0), 100 mM acetate buffer (pH = 4.0 and pH = 5.0), or neutral phosphate buffer (pH = 7.0) were mixed with the viruses. Following a 10-minute incubation period at 37°C, the mixtures underwent HA titration.

### 2.14. Cell Fusion Assay

Vero cells were infected with WT, WT-87G^+^, WT-295G^−^, or WT-87G^+^/295G^−^ at an MOI of 3, cultured for 16–24 hr, treated with 2 *μ*g/ml TPCK trypsin for 15 min, washed with PBS, and subsequently incubated in PBS at various pH values (pH: 5.0–6.0) for 10 min before being cultured in DMEM supplemented with 5% FBS for 3 hr. Then, 70% ethanol was added to fix the cells for 10 min, followed by Giemsa staining for 30 min to enable observation of cell fusion under a microscope.

### 2.15. Statistical Analyses

All the data were examined with GraphPad Prism software. Using Student's *t*-test, significant differences between various experimental groups were ascertained. At *P* < 0.05, differences were considered to be statistically significant.

## 3. Results

### 3.1. GMS on HA1 of H9N2 AIV

**Figure 1:**
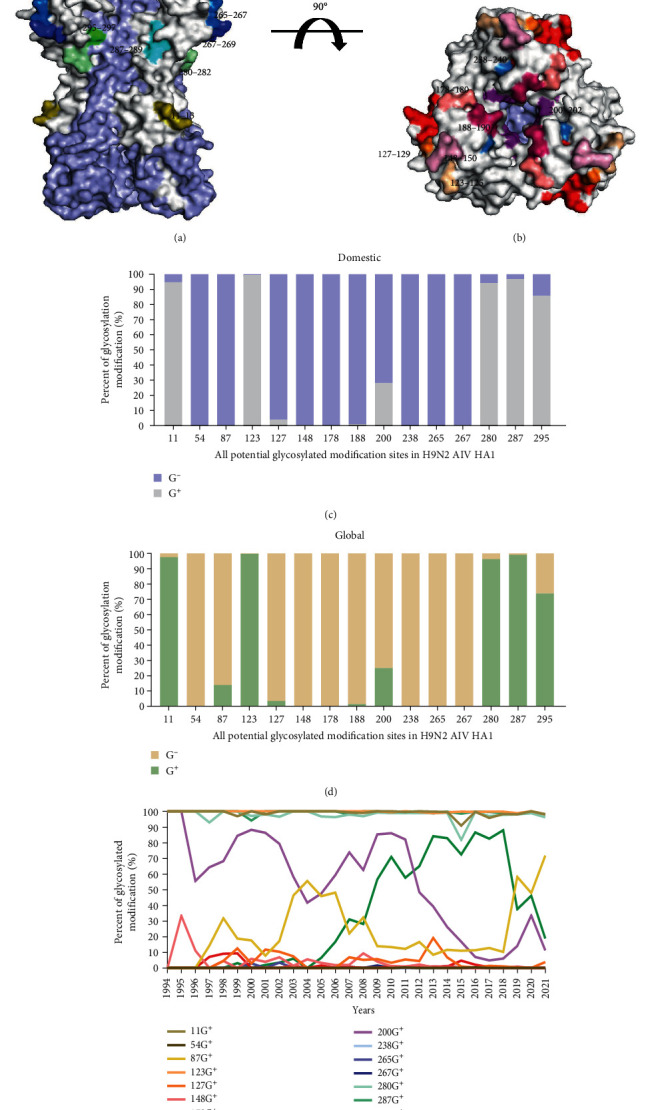
Three-dimensional (3D) structures and analysis of N-glycosylated modification sites in the HA trimer protein of H9N2 AIV. (a, b) HA trimer proteins are displayed from different angles. Different N-glycosylated modification sites are presented in different colors. The HA trimeric protein is divided into HA1 heads and HA2 stems. The HA1 head is represented in gray. The HA stem is represented in light blue. Olive labeling of GMS at positions 11–13, deep olive at 54–56, yellow at 87–89, wheat at 123–125, orange at 127–129, light pink at 148–150, salmon at 178–180, warm pink at 188–190, purple at 200–202, sky blue at 238–240, marine blue at 265–267, TV blue at 267–269, pale green at 280–282, cyan at 287–289, and green at 295–297. All H9N2 HA amino acid sequences of Chinese or worldwide isolates (as of December 2021) were downloaded from GISAID (https://www.gisaid.org) and aligned using MAFFT. After excluding homologous and duplicate sequences in BioEdit, the prevalence of each GMS was assessed. (c, d) Percent of all potential GMS from 1994 to 2021 domestically (light purple and gray) or globally (light yellow and light green). (e) The variation trend of every potential GMS from 1994 to 2021. The color of each potential GMS is the same as that of the 3D structure.

HA proteins have various GMSs located in different areas, such as the head or stem region. In addition, whether there are glycosylations at specific sites will vary over time, which affects the biological characteristics of the virus. As a result, we evaluated the distribution and statistical fraction of all possible GMS on HA1 of H9N2 AIV. The whole HA gene sequences of all H9N2 AIV strains from 1994 to 2021 were retrieved from the GISAID database (https://www.gisaid.org). By analyzing these sequences, it was discovered that a total of 15 potential GMSs had appeared on HA1 since the epidemic of H9N2 AIV. These sites were located at amino acid positions 11–13, 54−56, 87–89, 123−125, 127–129, 148−150, 178–180, 188−190, 200–202, 238−240, 265–267, 267−269, 280–282, 287−289, and 295–297 (H9 numbering). PyMOL was used to determine the exact locations of these sites on the HA trimer, as shown from different perspectives in Figures 1(a) and 1(b). Three sites (11–13, 280−282, and 295–297) were located at the stem region of HA, while the remaining nine were positioned in the head region. Additionally, 87–89, 127−129, and 178–180 were close to the receptor-binding region (RBS) of the head of HA.

By analyzing the glycosylation proportions of potential GMSs from 1994 to 2021, the fluctuation trends of both global and domestic potential GMS were found to be similar, with most of them being conservative. Most commonly, 11–13, 123−125, 280–282, and 287–289 were glycosylated, while 54–56, 87−89, 127–129, 148−150, 178–180, 188−190, 238–240, 265−267, and 267–269 were not glycosylated, as depicted in Figures 1(c) and 1(d). Detailed information is provided in Tables S1 and S2. As shown in Figure 1(e), 87–89, 200−202, and 295–297 exhibited considerable fluctuations over time. 87−89: The proportion of glycosylated products increased from 1996 to 2004, followed by a decrease from 2005 to 2018, but it has increased since 2019. 200−202: There is a degree of instability, and the overall trend is decreasing. 295−297: This number has shown an increasing trend since 2005. However, since 2019, it has been decreasing.

### 3.2. Generation of GMS Viruses

To explore the influence of different GMSs on virus antigenicity and pathogenicity, the initial step was to rescue different GMS viruses. We designed GMS in its opposite form to determine the impact of the mutation on the virus. We also speculated that 200G^+^/295G^−^ and other single-point superpositions would further affect the biological characteristics of the virus. Therefore, we designed the different GMS mutation forms shown in Table 1. Here, we used a 12-plasmid reverse genetics system to rescue different GMS viruses. The HA and NA genes were from A/Chicken/Jiangsu/875/2018, the internal genes were from A/Chicken/Jiangsu/875/2018, and the polymerase genes were from A/WSN/33(H1N1). All these genes were used to rescue WT. The HA gene of A/Chicken/Anhui/99/2017 was utilized as a pattern to carry out site-specific mutation to obtain HAs with diverse glycosylations, which were generated to rescue different GMS viruses. The glycosylation of recombinant viruses with particular mutation sites was successfully rescued, and the corresponding viral abbreviations are listed in Table 1. The HA and NA genes of the rescued viruses were extensively sequenced to confirm that no unexpected mutations occurred. WT was initially composed of a few GMSs, such as 11G^+^, 123G^+^, 280G^+^, 287G^+^, and 295G^+^. Rescued different GMS viruses included only 11 of the 15 GMSs, as viruses containing 11G^+^, 123G^+^, 238G^+^, and 287G^+^ could not be saved, or the virus titers were too low to conduct further experiments.

### 3.3. Antigenicity Analysis of GMS Viruses

The oligosaccharides present on the surface of the HA protein can impede the neutralizing effect of neutralizing antibodies on the virus, thus affecting its antigenicity. To explore the effects of various GMSs on antigenicity, we prepared different GMS viruses as inactivated oil emulsion vaccines to immunize chickens and subsequently isolated immune serum to perform cross-hemagglutination inhibition experiments [39].

**Figure 2:**
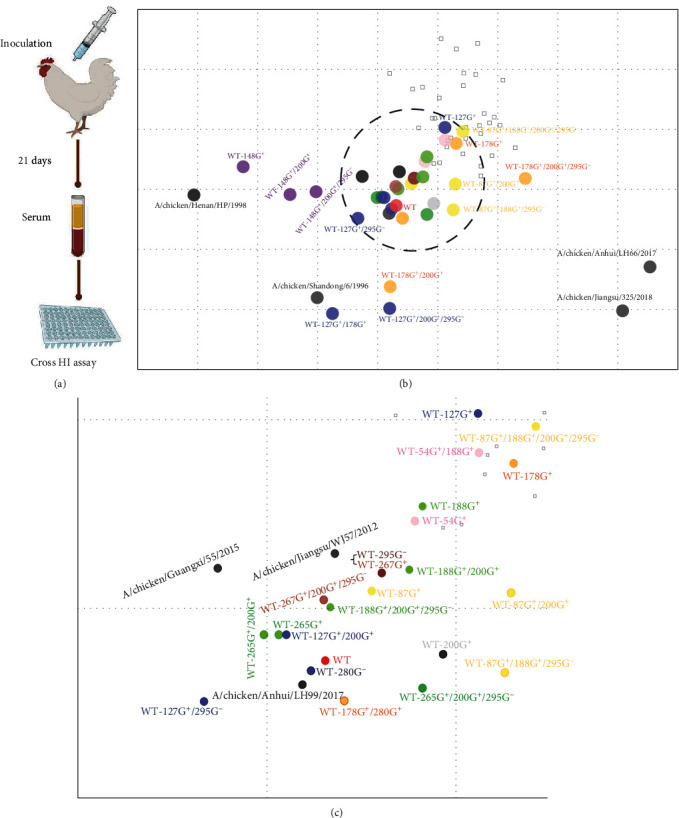
Analysis of the antigenicity of different GMS viruses. (a) Different GMS viruses were prepared as inactivated oil emulsion vaccines, and SPF chickens aged 4–6 weeks were immunized by intramuscular injection. Blood was collected 21 days after immunization, the serum was separated, and cross-hemagglutination inhibition experiments were subsequently conducted. The plants were created with https://BioRender.com. (b) Antigenic map of all GMS viruses. The scale bar in each map represents 1 antigenic unit (1 antigenic unit corresponds to a twofold dilution of antiserum in the HI assay). The map was produced by ACMACS (https://acmacs-web.antigenic-cartography.org/). Different GMS viruses (including site-associated combination viruses) are represented with different colors. WT: red, 54G^+^: pink, 87G^+^: yellow, 127G^+^: blue, 148G^+^: purple, 178G^+^: orange, 188G^+^: green, 200G^+^: gray, 265G^+^: forest green, 267G^+^: brown, 280G^−^: dark blue, and 295G^−^: dark brown. (c) Detailed antigenic map corresponding to the black-dotted bordered rectangle in Figure 2(b).

Figure 2(b) displays the antigen map, which was created based on the results of cross-hemagglutination inhibition experiments. We could assess the impact of various GMSs on the antigenicity of the virus by comparing the relative position between the GMS viruses and the WT on the antigen map. Upon analysis of the antigen map, the following could be observed: (1) All the mutant viruses showed a divergent state compared to WT, with the furthest antigenic distance from WT being roughly two cells. (2) 127G^+^, 148G^+^, and 178G^+^, which were near the RBS region, generated greater antigenic divergence. (3) Most GMSs (e.g., 87G^+^, 127G^+^, 148G^+^, 178G^+^, and 265G^+^) that were combined with 200G^+^/295G^−^ had greater antigenic divergence than those that were combined with 200G^+^ alone. (4) 127G^+^ and 178G^+^ (as well as their related viruses) caused the greatest differences on the map. (5) Single GMS viruses such as 54G^+^, 87G^+^, and 188G^+^ did not lead to a greater antigen distance than did WT. However, if 54G^+^ or 87G^+^ was combined with 188G^+^ (e.g., 54G^+^/188G^+^ and 87G^+^/188G^+^), the antigen distances on the map were greater.

### 3.4. Pathogenicity Analysis of GMS Viruses

Figure S1 thoroughly illustrates the comparison between the body weight changes, mortality, and viral titer of mouse lungs of a particular GMS and its related combination viruses. Figure S1(a, c, d, e, f) shows that 54G^+^, 127G^+^, 148G^+^, 178G^+^, and 188G^+^ could attenuate the pathogenicity of the WT strain in mice. However, 87G^+^, 265G^+^, 267G^+^, 280G^−^, and 295G^−^ may increase the pathogenicity of the WT strain in mice (Figure S1(b, g, h, i, j). Moreover, combining single GMS, such as 87G^+^, 127G^+^, 178G^+^, 188G^+^, 265G^+^, and 267G^+^, with 200G^+^/295G^−^, such as 127G^+^/200G^+^/295G^−^, 178G^+^/200G^+^/295G^−^, 265G^+^/200G^+^/295G^−^, and 267G^+^/200G^+^/295G^−^, resulted in greater pathogenicity in mice. Notably, 127G^+^/200G^+^/295G^−^, 148G^+^/200G^+^/295G^−^, 178G^+^/200G^+^/295G^−^, and 265G^+^/200G^+^/295G^−^ can create greater antigenic distances on the antigen map. In summary, 127G^+^/200G^+^/295G^−^, 178G^+^/200G^+^/295G^−^, and 265G^+^/200G^+^/295G^−^ had significant impacts on both pathogenicity and antigenicity.

**Figure 3:**
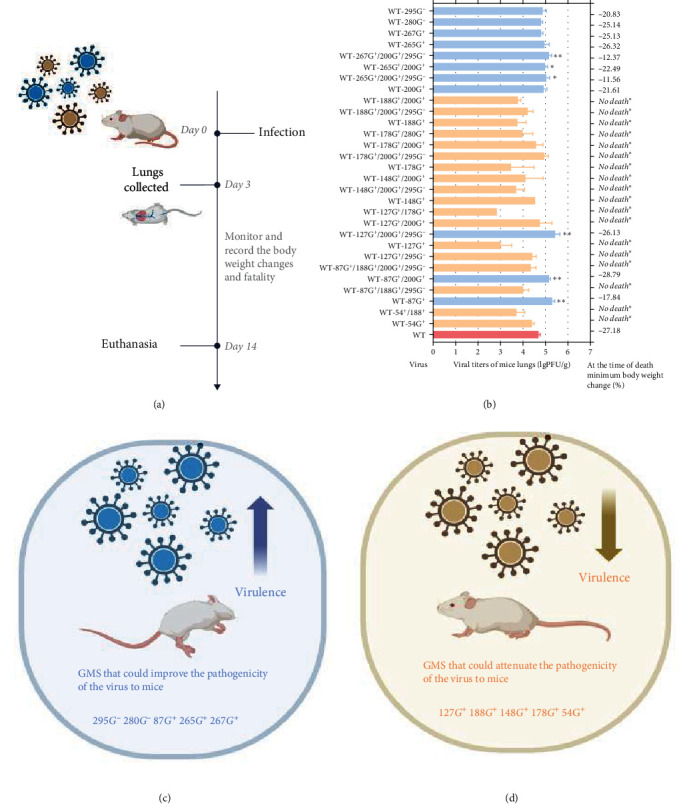
Pathogenicity of all GMS viruses in mice. (a) Mouse infection cartoon pattern diagram. Six-week-old SPF BALB/c mice were inoculated intranasally with 4 × 10^5^ PFU of all GMS viruses, changes in mouse body weight were continuously monitored until 14 days after challenge, and lungs were collected on day 3 postinfection for virus titration in MDCK cells. The plants were created with https://BioRender.com. (b) Viral titers in mouse lungs on day 3 postinfection with all GMS viruses and the minimum body weight change at the time of death. WT is represented in light pink. GMS viruses with stronger pathogenicity than WT viruses are represented in light blue. GMS viruses with weaker pathogenicity than WT viruses are represented in light yellow. Classification of GMS strains with different degrees of pathogenicity in mice. *No death* ^*∗*^ indicates that no mice died in this group. The values represent the means ± SDs from three independent experiments ( ^*∗*^*P<*0.05,  ^*∗∗*^*P<*0.01). (c) GMS could further enhance pathogenicity in mice (represented in light blue overall). The plants were created with https://BioRender.com. (d) GMS attenuated pathogenicity in mice (represented in light yellow overall). Source: Created with https://BioRender.com.

The glycosylation of the influenza virus HA protein is an important factor in determining viral pathogenicity. To assess the pathogenicity of different GMS viruses in mice, mice were intranasally inoculated with different GMS viruses at a concentration of 4 × 10^5^ PFU in 50 *μ*l. Taking into account the body weight change, mortality, and viral titer of mouse lungs, the pathogenicity of different GMS strains was comprehensively ranked. The most powerful GMS strains that could increase the pathogenicity of the virus to mice were 295G^−^, 280G^−^, 87G^+^, 265G^+^, 267G^+^, and 200G^+^. The GMSs that attenuated the pathogenicity of the virus to mice, from most powerful to least, were 127G^+^, 188G^+^, 148G^+^, 178G^+^, and 54G^+^ (Figures 3(c) and 3(d)). It is evident that the 87G^+^, 87G^+^/200G^+^, 127G^+^/200G^+^/295G^−^, 265G^+^/200G^+^/295G^−^, 265G^+^/200G^−^, and 267G^+^/200G^+^/295G^−^ groups had significantly greater virus titers in mouse lungs, as illustrated in Figure 3(b).

### 3.5. The Functional Glycosylation Sites Were at Positions 295–297, 280−282, and 87–89

**Figure 4:**
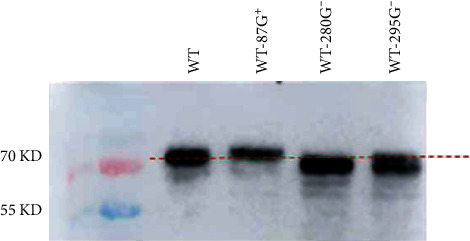
Western blotting to confirm N-linked glycosylation at the indicated sites on the mutated virus. WT, WT-87G^+^, WT-280G^−^, and WT-295G^−^ were used to infect MDCK cells at an MOI of 1. The infected cells were lysed to collect viral protein at 16 hr postinfection. Then, the viral protein was separated by 10% Tris SDS–PAGE, and anti-H9N2 HA mouse monoclonal antibodies were used as antibodies.

According to the results of the above pathogenicity experiments, three GMSs with the most significant effect on the pathogenicity of mice were selected: 295G^−^, 280G^−^, and 87G^+^. It is necessary to test whether these three GMSs are functional because these sites may not be glycosylated due to steric hindrance, and neighboring amino acids may further hinder glycan attachment [26]. Consequently, we explored the migration rates of the WT-295G^−^, WT-280G^−^, and WT-87G^+^ strains. As shown in Figure 4(a), compared with those of the WT, the migration rates of the 87G^+^ strain slightly decreased, while the migration rates of the 280G^−^ and 295G^−^ strains slightly increased. Consequently, the three selected GMSs were confirmed to be functional glycosylation sites. There are oligosaccharides attached to these positions that perform various functions.

To investigate whether there were any AIVs with other combinations of these three glycosylation sites in nature (87G^+^, 280G^−^, and 295G^−^) and whether viruses with a combination of these sites are more pathogenic to mammals, as WT-87G^+^, WT-280G^−^, and WT-295G^−^ individually were more pathogenic than WT. Based on these questions, upon analyzing the HA sequences of H9N2 AIV, it was discovered that there were three kinds of combinations of these three GMSs in nature: 87G^+^/295G^−^, 87G^+^/280G^−^/295G^−^, and 280G^−^/295G^−^ without 87G^+^/280G^−^. Then, we successfully rescued the WT-87G^+^/295G^−^, WT-87G^+^/280G^−^/295G^−^, and WT-280G^−^/295G^−^ strains and confirmed that there were no unwanted mutations. Their specific biological characteristics are listed in Table 2.

### 3.6. 87G^+^/295G^−^ Further Increased Pathogenicity, while 280G^−^/295G^−^ Decreased Pathogenicity

**Figure 5:**
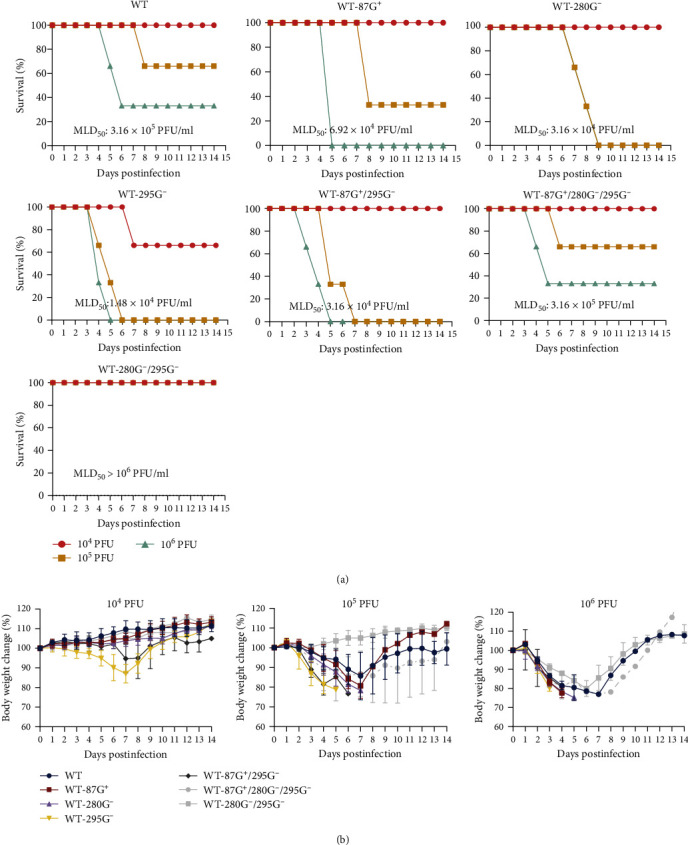
The effect of recombinant GMS viruses on the pathogenicity of mice. (a) Survival rate of mice infected with different GMS viruses; mean weight of mice infected with different GMS viruses (10^4^ PFU/50 *μ*l, 10^5^ PFU/50 *μ*l, or 10^6^ PFU/50 *μ*l) (*n* = 3). (b) The body weight change associated with every dose of the GMS virus. Mice were humanely euthanized when they lost ≥25% of their initial body weight.

To investigate whether recombinant viruses can further increase the pathogenicity of mice, the MLD_50_ values of these viruses were determined. According to the MLD_50_ values in Figure 5(a), the pathogenicity of the viruses to mice decreased in the following order: 295G^−^ > 87G^+^/295G^−^ = 280G^−^ > 87G^+^ > WT = 87G^+^/280G^−^/295G^−^ > 280G^−^/295G^−^. It has been determined that 280G^−^/295G^−^ does not increase the pathogenicity of the virus but instead reduces its pathogenicity. Moreover, compared with 280G^−^/295G^−^, 87G^+^/280G^−^/295G^−^ resulted in increased pathogenicity. Compared with 87G^+^, 87G^+^/295G^−^ had greater pathogenicity, while the MLD_50_ value of 87G^+^/295G^−^ was equal to that of 280G^−^.

As depicted in Figure 5(b), the body weight changes of the mice inoculated with these viruses showed that at 10^4^ PFU, the weight loss of the 295G^−^ group was significantly greater than that of the other groups. At 10^5^ PFU, the weight loss magnitude decreased in the following order: 295G^−^ > 87G^+^/295G^−^ > 280G^−^ > 87G^+^ > 87G^+^/280G^−^/295G^−^ > WT > 280G^−^/295G^−^. At 10^6^ PFU, the weight changes of the 87G^+^/295G^−^, 295G^−^, 87G^+^, and 280G^−^ groups were greater than those of the WT group, and the weight changes of the four groups could not be distinguished well, followed by those of the 87G^+^/280G^−^/295G^−^ and 280G^−^/295G^−^ groups. This experiment showed not only the effects of increased pathogenicity in mice by 87G^+^, 280G^−^, and 295G^−^ but also the greater pathogenicity when 87G^+^ and 295G^−^ were combined. Therefore, WT-87G^+^, WT-295G^−^, and WT-87G^+^/295G^−^ were chosen as the targets for further research.

### 3.7. 87G^+^ Impaired, but 295G^−^ Did Not Alter Human Receptor-Binding Properties

**Figure 6:**
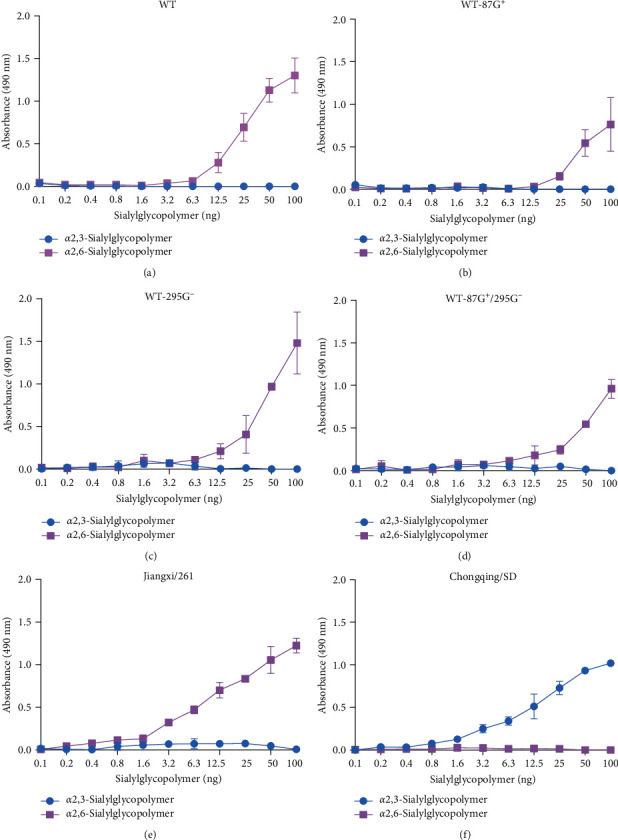
(a–f) Receptor-binding properties of different GMS viruses. The binding ability of different viruses to two different biotinylated glycans (*α*-2,3 glycan, blue; *α*-2,6 glycan, purple) was assessed. The data shown are the means of three repeats. The error bars indicate standard deviations.

Previous results showed that 87G^+^, 295G^−^, and 87G^+^/295G^−^ significantly enhanced the pathogenicity of the virus in mice, with 87 amino acids of the HA near the RBS region. We wanted to determine whether the increased pathogenicity of this virus is due to changes in its receptor-binding properties. We tested the receptor-binding properties of viruses with different GMSs. As shown in Figure 6, A/swine/Jiangxi/261/2016 (H1N1) and A/chicken/Chongqing/SD001/2021 (H5N6) were used as representative strains that significantly bind to human-derived *α*-2,6 sialic acid receptor and avian-derived *α*-2,3 sialic acid receptor, respectively. WT and three mutant viruses bind strongly to the *α*-2,6 sialic acid receptor. The binding patterns of WT and WT-295G^−^ to the *α*-2,6 sialic acid receptor were identical, indicating that 295G^−^ did not affect human receptor-binding capabilities. Compared with the WT strains, the WT-87G^+^ and WT-87G^+^/295G^−^ strains exhibited decreased affinity for the *α*-2,6 sialic acid receptor, demonstrating that 87G^+^ can alter the affinity of the virus for this receptor. Although 87G^+^ and 295G^−^ dramatically increased the virus's pathogenicity in mice, this was not due to a change in human receptor-binding capabilities. The affinity of the virus for the *α*-2,6 sialic acid receptor was dramatically reduced by 87G^+^ but not by 295G^−^.

### 3.8. 87G^+^ Decreased the Thermal and Acid Stability of the Virus, yet 295G^−^ Increased

When the HA protein is exposed to a neutral pH but the temperature continues to increase, its conformation shifts accordingly. HA protein stability refers to the maintenance of biological activity under various conditions, and it is a fundamental biological characteristic. A decrease in the acid and thermal stability of a virus is not beneficial for its survival in the external environment. However, after the virus invades the host cell, the decrease in acid stability (i.e., the increase in cell membrane fusion pH) will help the virus undergo membrane fusion as soon as possible and release the virus genome. Therefore, different viruses have optimal membrane fusion pH values to balance their activity in acidic extracellular environments and their acid sensitivity to membrane fusion in acidic endosomes [40].

**Figure 7:**
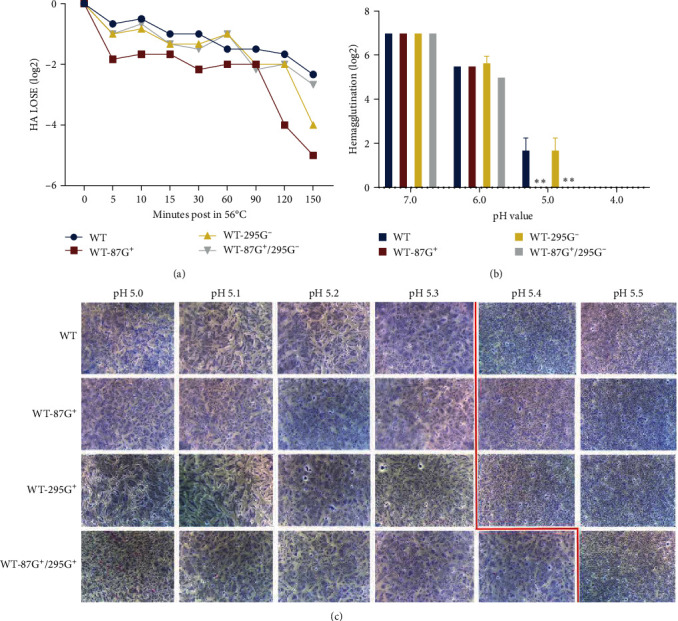
Effect of different GMS viruses on stability. (a) Effect of different GMS viruses on thermostability. The thermal stability was assessed by examining the ability of each virus to hemagglutinate chicken erythrocytes after incubation at 56°C over a time course. (b) Effect of different GMS viruses on pH stability. Seven log2 hemagglutination units (HAUs) of viruses were incubated in different buffers at 37°C for 10 min, and the viral titers were determined by the HA assay. The results are presented as log_2_ HA titers at the indicated pH values. The data are presented as the means ± SDs of results from three independent experiments. Statistical significance was determined by two-way ANOVA ( ^*∗∗*^*P* < 0.01). (c) Effect of different GMS viruses on membrane fusion ability. Syncytium formation in Vero cells infected with different GMS viruses at different pH values. The cells were fixed and stained with Giemsa solution. The red lines represent the range of pH values at which fusion was detected microscopically.

To investigate whether glycosylation affects thermal stability, the decreases in the HA titer of different GMS viruses were analyzed. As shown in Figure 7(a), 87G^+^ had the poorest thermal stability, followed by 295G^−^ and 87G^+^/295G^−^, and WT had the greatest thermal stability. To investigate whether the recombinant virus affects acid stability, the hemagglutination titer was then measured, as shown in Figure 7(b), at pH 4, 6, and 7. There was no significant difference in the hemagglutination values. However, at pH 5, the HA titers of 87G^+^ and 87G^+^/295G^−^ decreased significantly. At a pH of 6, the hemagglutination titer of the WT-295G^−^ strain was slightly greater than that of the WT strain. The introduction of 87G^+^ or 87G^+^/295G^−^ to the HA of the WT strain decreased the acid stability of the virus. In the absence of glycosylation at position 295, the acid stability of the virus slightly increased. Therefore, it was speculated that the decrease in acid stability of 87G^+^/295G^−^ was largely caused by 87G^+^ rather than 295G^−^.

Membrane fusion occurs after the virus infects host cells due to a conformational shift in the HA protein produced by the acidity of the capsule membrane. When the syncytium formed, multiple Vero cells underwent membrane fusion and fusion, resulting in nuclear aggregation and fusion, and large cell clusters with blue Giemsa staining could be observed. If a syncytium cannot form, Vero cells cannot undergo membrane fusion, thus maintaining their individual dispersed cell state. After staining, the dispersed single nuclei were evenly distributed [41, 42]. To determine the effect of the four recombinant viruses on cell fusion, syncytial formation experiments were conducted. The results are shown in Figure 7(c). At pH = 5.3, syncytium formation in the WT, 87G^+^, and 295G^−^ strains was observed; however, the degree to which the syncytium formed in the WT and 87G^+^ strains was greater than that in the 295G^−^ strain, while the pH at which the syncytium formed in the 87G^+^/295G^−^ strain was 5.4, which increased the pH of cell membrane fusion. The lower the pH of membrane fusion, the better the acid stability. Therefore, the acid stability of the WT-295G^−^ mutant was slightly better than that of WT, while the acid stability of the WT-87G^+^/295G^−^ mutant was worse than that of WT. Overall, 295G^−^ slightly improved the effect on viral acid stability. Moreover, 87G^+^ significantly weakened the stability of the virus.

### 3.9. 87G^+^, 295G^−^, and 87G^+^/295G^−^ Could Further Enhance the Inflammatory Response of Infected Mouse Lungs

**Figure 8:**
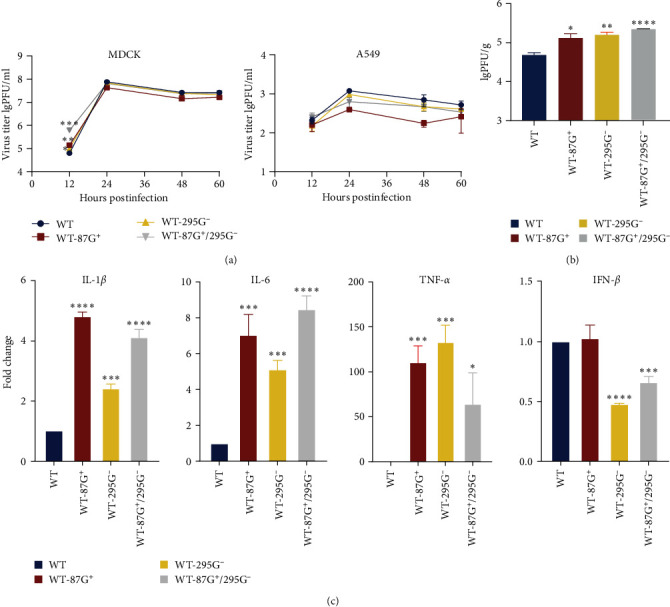
The viral replication of different GMS viruses *in vitro* and *in vivo*. (a) Growth kinetics of different GMS viruses in MDCK and A549 cells. Growth curves of different cell lines infected with WT, WT-87G^+^, WT-280G^−^, or WT-295G were generated at multiplicities of infection (MOIs) of 0.01 and 0.5. Then, cell supernatants were collected at 12, 24, 36, and 48 hr postinfection, and viral titers were determined via plaque assays. The values represent the means ± SDs from three independent experiments ( ^*∗*^*P* < 0.05,  ^*∗∗*^*P* < 0.01, and  ^*∗∗∗*^*P* < 0.001). (b) Viral titers in mouse lungs on day 3 postinfection. Six-week-old SPF BALB/c mice were inoculated intranasally with 1 × 10^5^ PFU of WT, WT-87G^+^, WT-280G^−^, or WT-295G^−^, and lungs were collected on day 3 postinfection for virus titration in MDCK cells by plaque assay ( ^*∗*^*P* < 0.05,  ^*∗∗*^*P* < 0.01,  ^*∗∗∗*^*P* < 0.001, and  ^*∗∗∗∗*^*P* < 0.0001). (c) Cytokine production in the lungs of mice. Lungs were collected on day 3 postinfection. Relative mRNA levels of IL-1*β*, IL-6, TNF-*α*, and IFN-*β* in infected mouse lungs were measured by RT-qPCR. All values were normalized to GAPDH and are expressed as the fold change compared with controls. The values represent the means ± SDs from three independent experiments ( ^*∗*^*P* < 0.05,  ^*∗∗*^*P* < 0.01,  ^*∗∗∗*^*P* < 0.001, and  ^*∗∗∗∗*^*P* < 0.0001).

To analyze the virus replication efficiency *in vitro*, MDCK and A549 cells were infected with the modified virus, and the cell supernatants were collected for titration. The results are shown in Figure 8(a). The replication titers of the three modified viruses were greater in the MDCK cells 12 hr after infection than in the WT cells. However, except for the WT-87G^+^ strain, whose replication titer was lower than that of the WT strain, there was no difference in replication titer between the WT-295G^−^, WT-87G^+^/295G^−^, and WT strains. In the A549 cells, the replication titers of the three mutated viruses were lower than those of the WT strain at any time point. The results indicated that the replication titers of the four mutated viruses in MDCK cells were indistinguishable, while those in A549 cells were low.

To detect the replication titers of the virus in mice and the expression levels of cytokines in the lungs, on the third day after inoculation, the mice were killed, and their lungs were collected for viral titration of MDCK cells.  Figure 8(b) shows that the replication titers of the three mutated viruses were much greater than those of the WT virus, demonstrating that the mutated viruses increased replication in the mouse lungs. Next, we examined cytokine levels in the lungs of the mice. IL-1*β*, IL-6, and TNF-*α* are proinflammatory cytokines, while IFN-*β* is an anti-inflammatory cytokine. The expression levels of cytokines in the lungs were detected by RT-qPCR, as shown in Figure 8(c). The three mutant viruses showed considerably greater expression levels of IL-1*β*, IL-6, and TNF-*α* than did the wildtype. The expression of IFN-*β* in the WT-87G^+^ strain was similar to that in the WT strain, yet the expression of IFN-*β* in the other two viruses was significantly decreased. Therefore, infecting mice with three different mutated viruses, 87G^+^, 295G^−^ and 87G^+^/295G^−^, could result in a greater inflammatory response than that in WT mice, thus leading to more extensive lung damage.

## 4. Discussion

The glycosylation of the influenza virus HA protein is thought to be a method of masking or covering antibody-binding sites, allowing immunological escape. As a result, certain GMSs in the head of the HA protein are bound to impact the antigenicity of the virus [29, 43, 44]. Analysis of the antigen map revealed that the combination of 200G^+^/295G^−^ could increase the antigen distance on the antigen map. It has also been reported that immunization with a 200G^+^/295G^−^ combined vaccine in chickens can provide 100% protection against 200G^+^/295G^+^ and 200G^−^/295G^+^ virus reinfection [45]. However, the 200G^+^/295G^−^ epidemic proportion has decreased in the last few years. When two or more GMSs were combined, such as 54G^+^/188G^+^ and 87G^+^/188G^+^, the virus was able to cause greater antigenic divergence simultaneously because of the masking effect of glycosylation on the HA protein, making it more difficult for antibodies to neutralize. It has been documented that antibodies derived from vaccines prepared with viruses modified with more glycosylated sites have a wider range of neutralizing activity than antibodies derived from viruses modified with fewer glycosylated sites [30], suggesting that viruses with more glycosylations of HA as vaccine candidates can provide cross-protective advantages for different viral strains [29]. The HA protein of seasonal H1N1 subtype influenza viruses acquired two additional glycosylation sites at positions 129 and 163 (H9: positions 127 and 156), providing viruses with neutralizing activity against antisera produced by any of the wild-type viruses [27]. The introduction of new oligosaccharides at positions 63, 122, 126, 133, and 246 (H9: positions 56, 115, 121, 127, and 236) of the HA protein of the H3N2 subtype influenza virus leads to immune escape by altering its antigenicity [32]. Antigenicity research revealed that 127G^+^ and its related viruses could generate greater antigenic divergence. In addition, 127G^+^ and 178G^+^ (as well as their related viruses) caused the greatest distance on the map. It has been shown that H9N2 AIV demonstrated the most prominent antigen escape due to 127 and 183 amino acid changes in the HA gene, resulting in an oligosaccharide connected at residue 127 [22]. During the evolution of human seasonal influenza viruses, the number of GMS on the HA protein has been increasing annually [27], showing a selective advantage, and an increase in GMS can effectively prevent the binding of neutralizing antibodies to antigenic epitopes [46]. As a result, the acquisition of potential GMS is a successful approach for influenza viruses to avoid host immune pressure [35].

In this study, the sites with enhanced pathogenicity in mice were mainly located at the edge of HA, for example, 295G^−^, 280G^−^, 87G^+^, 265G^+^, and 267G^+^. The areas of decreased pathogenicity in the H1N1 subtype influenza virus were also localized at the margin of the HA head at positions 71G^+^ and 286G^+^ (H9: positions 71 and 280) [21]. We also found that viruses with 127G^+^, 188G^+^, 148G^+^, 178G^+^, or 54G^+^ could attenuate pathogenicity in mice. The addition of GMS at position 127 of the HA protein of H9N2 AIV reportedly diminishes the pathogenicity of the virus in mice, which is consistent with our research results [22]. Although 87G^+^, 295G^−^, and 87G^+^/295G^−^ could increase pathogenicity in mice, the replication titers of the three recombinant mutated viruses were all greater than those of WT in the first 12 hr in MDCK; however, after 12 hr, the replication titers of the four viruses were nearly indistinguishable. In A549 cells, the WT replication titer was always greater than that of the three recombinant mutant viruses. It has been reported that when GMS at positions 10, 23, and 286 (H9: positions 10, 23, and 280) on HA of the H5N1 subtype influenza virus is removed, the cleavage of HA is almost completely blocked, leading to a significant decrease in the growth rates of the mutant viruses in MDCK and CEF cells [47]. In comparison to the wild-type virus of the H5N1 subtype influenza virus, viruses that lack glycosylation at either position 158 or 169 (H9: positions 152 and 163) had a significantly lower growth titer in both MDCK and A549 cells but displayed increased pathogenicity in mice [21]. The pathogenicity of the virus is sometimes not positively correlated with its replication efficiency in specific cells.

IL-1*β*, IL-6, and TNF-*α* are proinflammatory factors. In this study, infection of mice with WT-87G^+^, WT-295G^−^, or WT-87G^+^/295G^−^ significantly increased the expression levels of proinflammatory factors in their lungs, resulting in severe inflammatory reactions. Severe inflammatory reactions in mouse lungs are often associated with high pathogenicity of the virus. It was reported that the removal of glycosylation at positions 158 or 169 (H9: positions 152 and 163) of the H5N1 subtype of avian influenza virus could increase the pathogenicity of the virus to mice, and the expression levels of cytokines such as IL-6, IL-8, and MX-1 in mouse lungs are significantly increased [48]. The addition of glycosylation at position 158 (H9: position 152) of the H5N6 subtype of avian influenza virus can enhance the pathogenicity of the virus to mice, resulting in increased levels of inflammatory factors in mouse lungs (e.g., HMGB1, IL-10, and TNF-*α*) [49]. Mice infected with WT-295G or WT-87G^+^/295G showed decreased expression of the antiviral cytokine IFN-*β* in their lungs. It has been reported that the expression level of IFN-*β* detected in some elderly individuals infected with the influenza virus is significantly reduced compared to that in young people to prevent the immune system from detecting the virus's genes, allowing the virus to replicate unchecked and ultimately causing severe inflammatory reactions [50].

SP-D is a mouse lung surface protein belonging to one of the lectins in the collectin family [51]. It tends to bind oligosaccharides that end in mannose [52]. SP-D binds with the high mannose−oligosaccharide chains of the HA and NA proteins of the influenza virus, thus inhibiting their activity, resulting in viral aggregation and decreased infectivity [53, 54]. Several studies have shown that the absence of glycosylation at sites of the influenza virus HA protein, such as residue 158 (H9: position 152) of the H5N1 subtype, can cause increased pathogenicity in mice [21]. The greater pathogenicity observed due to the deletion of GMS is attributed to the reduced sensitivity to SP-D. A/Hong Kong 1/68 (H3N2) HA was changed with several GMSs, resulting in greater susceptibility to SP-D and lower pathogenicity in mice [34]. The removal of glycosylated HA1 from the H3 subtype (A/Beijing/353/89) and H1 subtype (A/Brazil/11/78) reduced the neutralization of the virus by SP-D, resulting in enhanced pathogenicity in mice [55, 56]. Our findings imply that 295G^−^ and 280G^−^ can increase viral pathogenicity in mice. Therefore, we believe that the greater pathogenicity in mice is likely due to the loss of GMS, which causes decreased sensitivity to SP-D; however, this hypothesis was not examined in detail in this research and requires further evidence.

## 5. Conclusions

We generated viruses modified with various GMSs to explore the effects of GMS on antigenicity and pathogenicity. Antigenic analysis revealed that viruses with 200G^+^/295G^−^ combined with single GMS, such as 87G^+^, 127G^+^, 148G^+^, 178G^+^, and 265G^+^, could significantly affect the antigenicity of the virus. Pathogenicity analysis revealed that the addition of GMS, such as 127G^+^, 188G^+^, 148G^+^, 178G^+^, or 54G^+^, decreased the virulence of the virus, except for 87G^+^. The removal of GMS, such as 280G^−^ or 295G^−^, increased the virulence of the virus in mice. Further pathogenicity studies revealed that 87G^+^/295G^−^ could also enhance the pathogenicity of the virus, while 280G^−^/295G^−^ weakened it. This study can guide epidemiologic surveillance results, provide early warning of cross-host adaptation of H9N2 AIV, and reveal the potential threat to public health security.

## Figures and Tables

**Table 1:** Detailed information on different GMS viruses.

Serial number	Detailed glycosylated site added or deleted under WT background	Hemagglutinin titer (log2)	Virus titer (PFU/ml)
WT	(11G^+^, 123G^+^, 280G^+^, 287G^+^, and 295G^+^)^*a*,*b*^	11	8.5 × 10^8^
R-1	WT-54G^+^	11	5 × 10^8^
R-2	WT-54G^+^/188G^+^	10	4.15 × 10^8^
R-3	WT-87G^+^	9	9.5 × 10^7^
R-4	WT-87G^+^/188G^+^/295G^−^	11	2.05 × 10^8^
R-5	WT-87G^+^/200G^+^	8	2.05 × 10^7^
R-6	WT-87G^+^/188G^+^/200G^+^/295G^−^	12	7.5 × 10^8^
R-7	WT-127G^+^/295G^−^	10	1.15 × 10^8^
R-8	WT-127G^+^	10	5.5 × 10^8^
R-9	WT-127G^+^/200G^+^/295G^−^	10	2.6 × 10^8^
R-10	WT-127G^+^/200G^+^	10	1.25 × 10^8^
R-11	WT-127G^+^/178G^+^	10	1.05 × 10^9^
R-12	WT-148G^+^	10	6.5 × 10^8^
R-13	WT-148G^+^/200G^+^/295G^−^	9	1.45 × 10^9^
R-14	WT-148G^+^/200G^+^	10	1.1 × 10^9^
R-15	WT-178G^+^	11	9.5 × 10^8^
R-16	WT-178G^+^/200G^+^/295G^−^	12	1.15 × 10^9^
R-17	WT-178G^+^/200G^+^	11	4.7 × 10^8^
R-18	WT-178G^+^/280G^+^	11	4.15 × 10^8^
R-19	WT-188G^+^	11	8 × 10^8^
R-20	WT-188G^+^/200G^+^/295G^−^	12	1.95 × 10^9^
R-21	WT-188G^+^/200G^+^	10	2.65 × 10^8^
R-22	WT-200G^+^	11	8 × 10^8^
R-23	WT-265G^+^/200G^+^/295G^−^	10	2.5 × 10^8^
R-24	WT-265G^+^/200G^+^	12	7.5 × 10^8^
R-25	WT-267G^+^/200G^+^/295G^−^	11	2.65 × 10^8^
R-26	WT-265G^+^	11	7 × 10^8^
R-27	WT-267G^+^	11	8 × 10^8^
R-28	WT-280G^−^	10	1.95 × 10^8^
R-29	WT-295G^−^	11	2.65 × 10^8^

^*a*^Position numbers are according to H9 numbering. ^*b*^The WT protein originally contains four N-glycosylated sites, 11G^+^, 123G^+^, 280G^+^, 287G^+^, and 295G^+^.

**Table 2:** The basis of GMS combination viruses.

Serial number	Detailed glycosylated site added or deleted under WT background	Hemagglutinin titer (log2)	Virus titer(PFU/ml)
WT	(11G^+^, 123G^+^, 280G^+^, 287G^+^, and 295G^+^)^*a*,*b*^	11	8.5 × 10^8^
R-30	WT-87G^+^/295G^−^	10	6.05 × 10^8^
R-31	WT-87G^+^/280G^−^/295G^−^	9	1.88 × 10^8^
R-32	WT-280G^−^/295G^−^	10	3.8 × 10^8^

^*a*^Position numbers are according to H9 numbering. ^*b*^The WT protein originally contains four N-glycosylated sites, 11G^+^, 123G^+^, 280G^+^, 287G^+^, and 295G^+^.

## Data Availability

The data supporting the study's findings are available from the corresponding author upon request.

## Supplementary Materials

Supplementary Materials Table S1: detailed information about the proportions of glycosylations of different GMSs globally. Table S2: detailed information about the proportions of domestic glycosylations of different GMSs. Table S3: detailed primers sequences information about specific GMS. Table S4: primers of quantitative real-time RT-PCR assay. Figure S1: body weight changes, viral titers in the lungs, and fatality rates of each group of GMS viruses.
