# Supplementary material for: Effects of the Glycosylation of the HA Protein of H9N2 Subtype Avian Influenza Virus on the Pathogenicity in Mice and Antigenicity
Source: Transbound Emerg Dis. 2024 May 17;2024:6641285. doi: 10.1155/2024/6641285 (PMC12016912; doi:10.1155/2024/6641285)
Supplement: Supplementary Materials — Table S1: detailed information about the proportions of glycosylations of different GMSs globally. Table S2: detailed information about the proportions of domestic glycosylations of different GMSs. Table S3: detailed primers sequences information about specific GMS. Table S4: primers of quantitative real-time RT-PCR assay. Figure S1: body weight changes, viral titers in the lungs, and fatality rates of each group of GMS viruses. [file 6641285.f1.pdf]

Table S1 Detailed information about the proportion of glycosylation modification of different GMS globally.

	HA1														
Glycosylation forms	NSTE	NCTI	NGTC	NVSY/ NVTY	NGTS	NNTY/ NNSY/ NGSY	NTTQ/ NDTQ	NDTT/ NDTS	NRTF	NGSL	NGSC	NCTV/ NSSG	NTTL/ NSTL	NVSK/ NISK/N VSR	NCSK
Glycosylated sites	11-13	54-56	87-89	123-125	127-129	148-150	178-180	188-190	200-202	238-240	265-267	267-269	280-282	287-289	295-297
Number of glycosylated modification	7691	5	1097	7865	269	48	19	109	1968	2	2	23	7588	7819	5821
The proportion of glycosylation modification(total number:7888)	97.50%	0.06%	13.91%	99.71%	3.41%	0.61%	0.24%	1.38%	24.95%	0.03%	0.03%	0.29%	96.20%	99.13%	73.80%

Table S2 Detailed information about the proportion of glycosylation modification of different GMS in domestic.

	HA1														
Glycosylation forms	NSTE	NCTI	NGTC	NVSY/ NVTY	NGTS	NNTV	NTTD	NDTT	NRTF	NGSL	NGSC	NCTV	NTTL	NVSK/ NISK	NCSK
Glycosylated sites	11-13	54-56	87-89	123-125	127-129	148-150	178-180	188-190	200-202	238-240	265-267	267-269	280-282	287-289	295-297
Number of glycosylated modification	6182	6	10	6486	257	20	14	70	1837	2	3	24	6140	6317	5590
The proportion of glycosylation modification(total number:6519)	94.83%	0.09%	0.15%	99.49%	3.94%	0.31%	0.21%	1.07%	28.18%	0.03%	0.05%	0.37%	94.19%	96.90%	85.75%

Downloaded the HA sequences from 1994 to 2021 of H9N2 avian influenza virus globally or in domestic from GISAID database (https://www.gisaid.org). There were 7888 sequences globally and 6519 sequences in domestic, and domestic sequences were included in the global sequences. Summarized the glycosylation modification proportion of different GMS, as illustrated in Table S1 and Table S2.

Table S3 Detailed primers sequences information about specific GMS

	Amino acid position	Primers used	Primers sequences(5'-3')
Removal of the GMS	11-13	F	AATCAACAAACTCCGCGGAAACTGTGGACACACT
		R	AGTGTGTCCACAGTTTCCGCGGAGTTTGTTGATT
	123-125	F	ACACAATCTGGAGTGTGACTTACAGTGGGACAAGC
		R	GCTTGTCCCACTGTAAGTCACACTCCAGATTGTGT
	280-282	F	GAAAGGTGGCTTAAACACAATATTGCCCTTCCAAAATG
		R	CATTTTGGAAGGGCAATATTGTGTTTAAGCCACCTTTC
	287-289	F	AACATTGCCCTTCCAAAAGGTAAGTAAGTATGCATTTG
		R	CAAATGCATACTTACTTACCTTTTGGAAGGGCAATGTT
	295-297	F	GCATTTGGAAACTGCCCAAAGTACATTGGTGTA
		R	TACACCAATGTACTTTGGGCAGTTTCCAAATGC
Addition of the GMS	54-56	F	CTCTTATTCTAGACAACTGCACCATTGAAGGGC
		R	GCCCTTCAATGGTGCAGTTGTCTAGAATAAGAG
	87-89	F	TCAGCTGTTAACGGAACGTGTTATCCCGGGAATGT
		R	ACATTCCCGGGATAACAGTATCCGTTAACAGCTGA
	127-129	F	TGGAATGTGTCTTACAATGGGACAAGCAAAGC
		R	GCTTTGCTTGTCCCATTGTAAGACACATTCCA
	148-150	F	TCAAAAGAACAACACCTACCCAACCCAAGATGCT
		R	AGCATCTTGGGTTGGGTAGGTGTTGTTCTTTTGA
	178-180	F	AAATCACCCACCCACCAATACTACGCAGACAA
		R	TTGTCTGCGTAGTATTGGTGGGTGGGTGATTT
	188-190	F	AAATCTGTACACCAGAAACGACACAACAACGA
		R	TCGTTGTTGTGTCGTTTCTGGTGTACAGATTT
	200-202	F	GGAAATGAATAGGACCTTTAAACCATTGATAG
		R	CTATCAATGGTTTAAAGGTCCTATTCATTTCC
	238-240	F	ACTGCGGATAAAATCTAATGGGAGTCTAATAGCTCCAT
		R	ATGGAGCTATTAGACTCCCACCAGATTTTATCCGCAGT

Table S4 Primers of quantitative real-time RT-PCR assay

Cytokines	Primers used	Primers sequences(5'-3')
IL-1β	F	GAAATGCCACCTTTTGACAGTG
	R	TGGATGCTCTCATCAGGACAG
IL-6	F	CTGCAAGAGACTTCCATCCAG
	R	AGTGGTATAGACAGGTCTGTTGG
TNF-α	F	CCTGTAGCCCACGTCGTAG
	R	GGGAGTAGACAAGGTACAACCC
IFN-β	F	TGGGTGGAATGAGACTATTGTTG
	R	CTCCCACGTCAATCTTTCCTC
GAPDH	F	TGACCTCAACTACATGGTCTACA
	R	CTTCCCATTCTCGGCCTTG

Figure S1

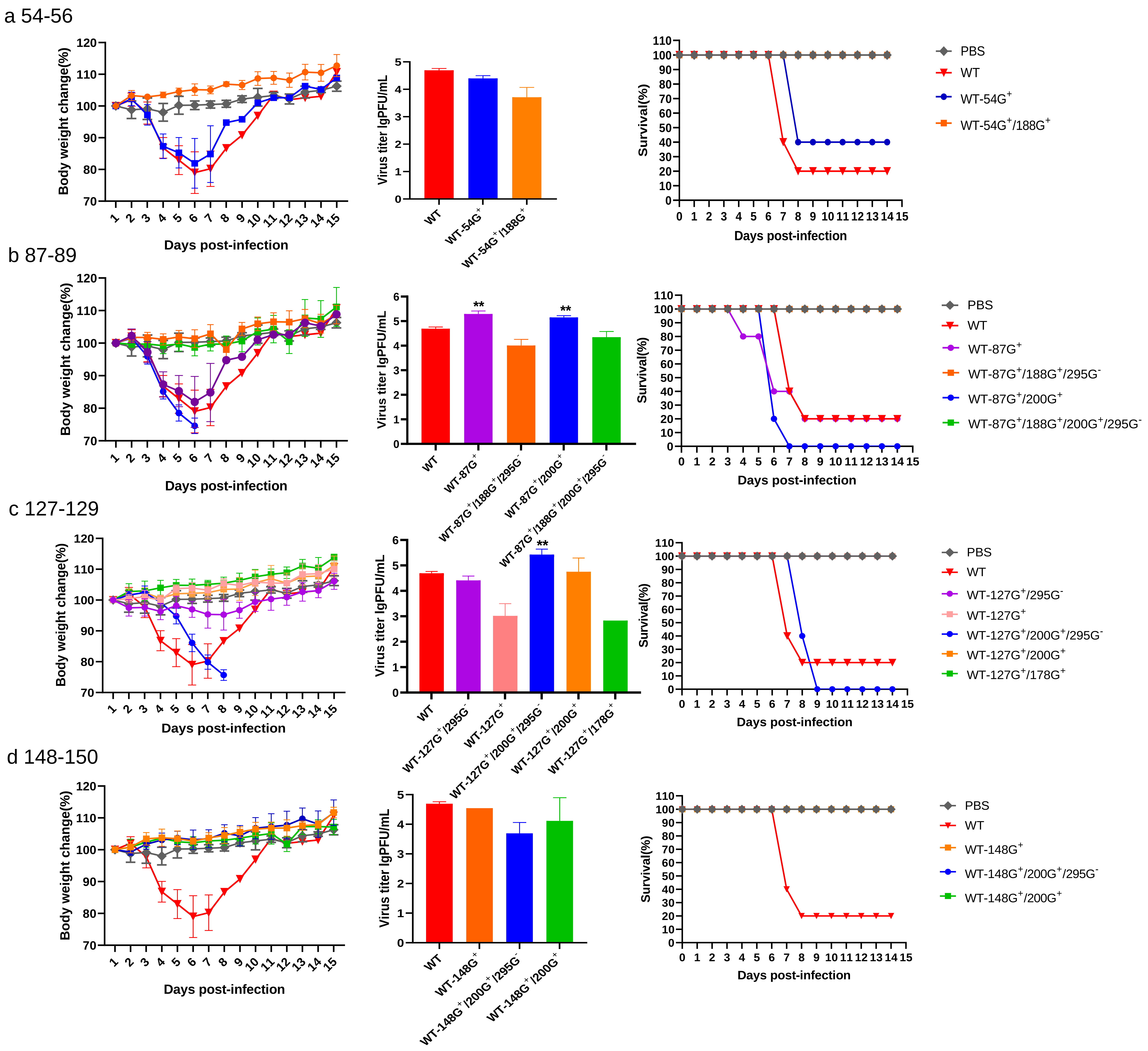
a 54-56
b 87-89
c 127-129
d 148-150
Body weight change(%)
Days post-infection
Virus titer lgPFU/mL
Survival(%)
**
PBS
WT
WT-54G+
WT-54G+/188G+
WT-87G+
WT-87G+/188G+/295G-
WT-87G+/200G+
WT-87G+/188G+/200G+/295G-
WT-127G+/295G-
WT-127G+
WT-127G+/200G+/295G-
WT-127G+/200G+
WT-127G+/178G+
WT-148G+
WT-148G+/200G+/295G-
WT-148G+/200G+

Figure S1

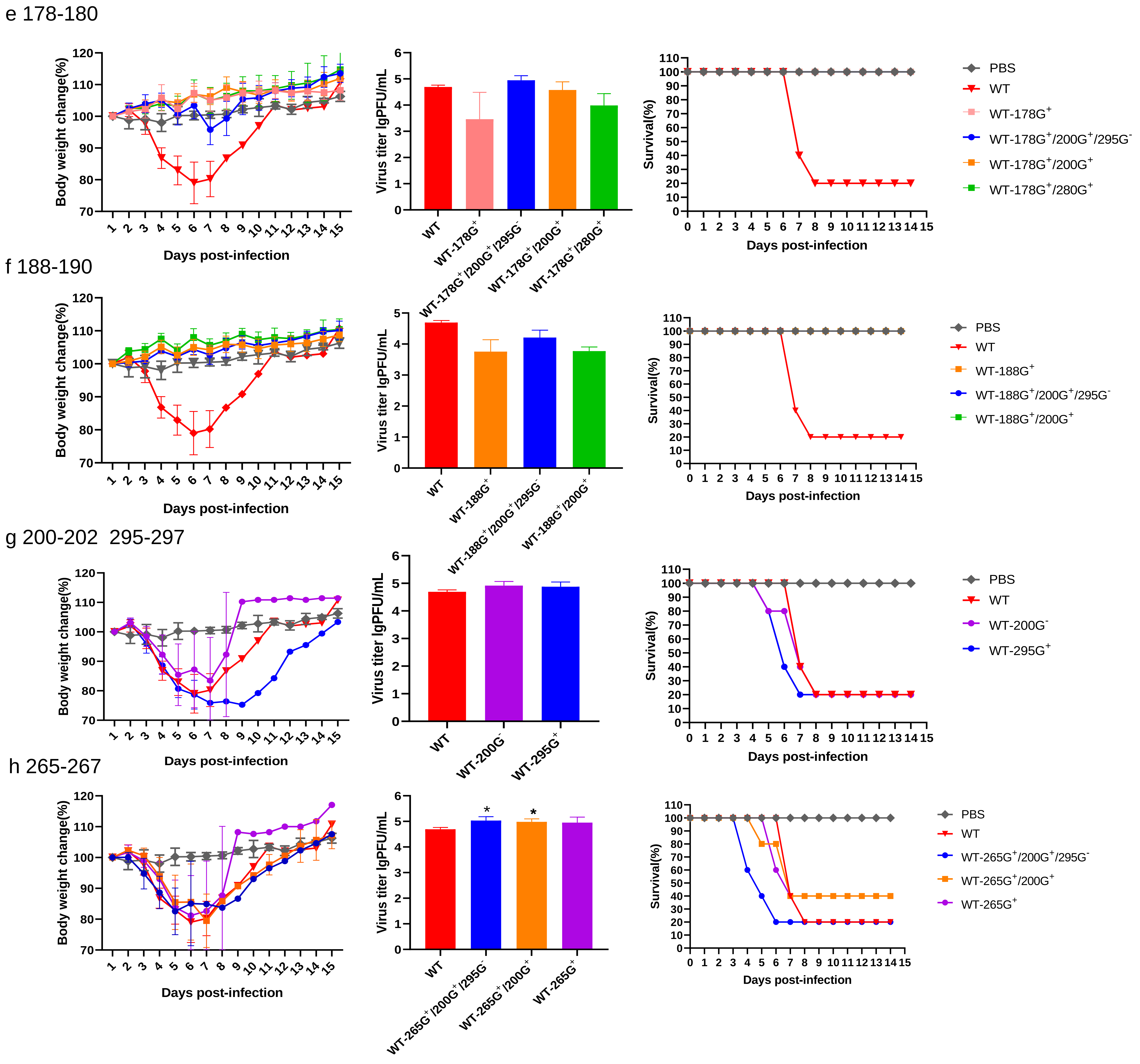
e 178-180
Body weight change(%)
Days post-infection
Virus titer lgPFU/mL
WT
WT-178G+
WT-178G+/200G+/295G-
WT-178G+/200G+
WT-178G+/280G+
Survival(%)
PBS
f 188-190
WT-188G+
WT-188G+/200G+/295G-
WT-188G+/200G+
g 200-202 295-297
WT-200G-
WT-295G+
h 265-267
WT-265G+/200G+/295G-
WT-265G+/200G+
WT-265G+
*

Figure S1

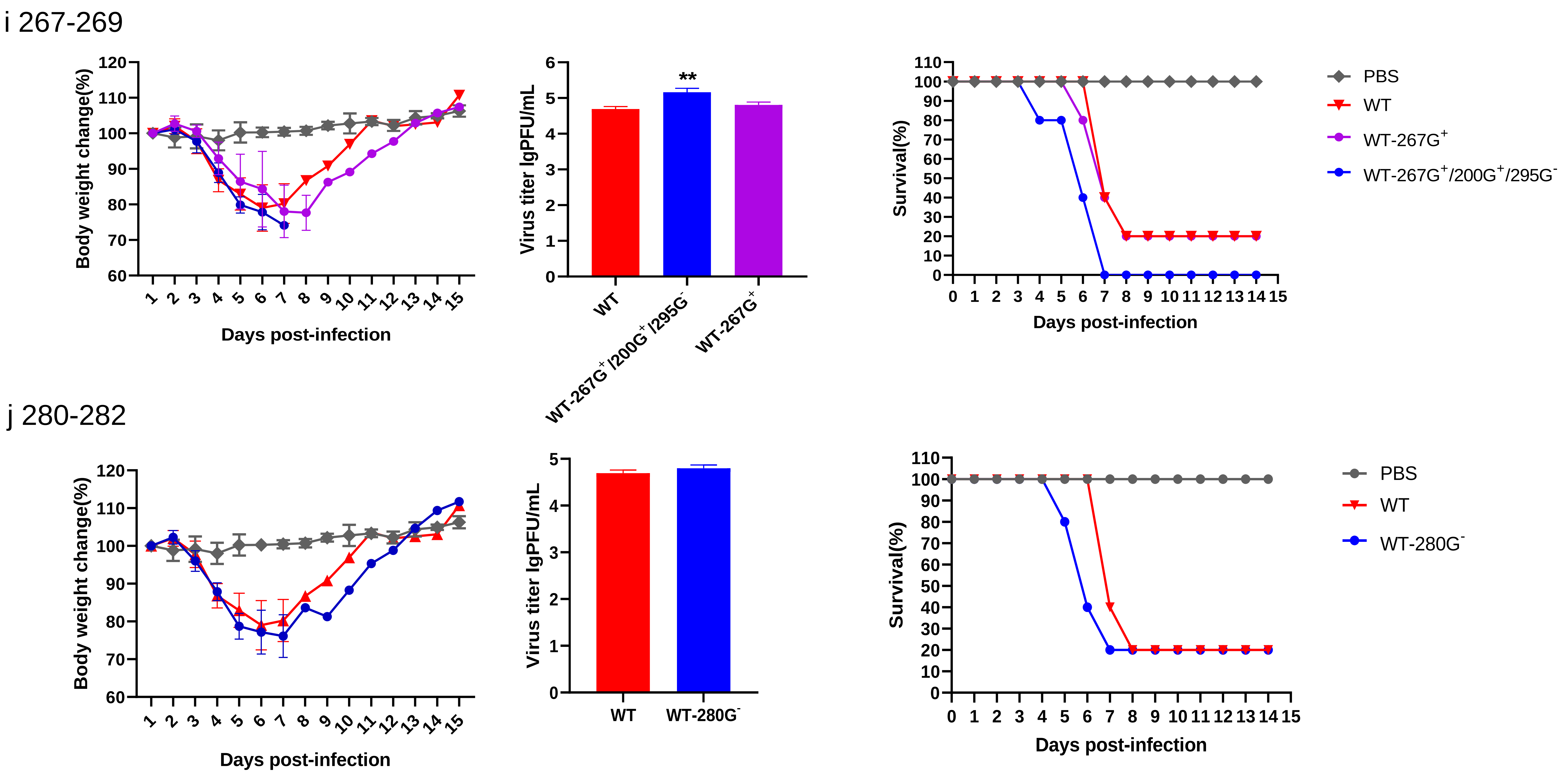

Figure S1: The body weight change, viral titers of lungs and fatality rate of every group of GMS viruses. Six-week-old SPF Balb/c mice were inoculated intranasally with 4×10^5 PFU all GMS viruses, continuously monitored changes in mouse body weight until 14 days after challenge and lungs were collected on day 3 post-infection for virus titration in MDCK cells. Different letters from a to j represented different GMS groups, every group contained single site GMS virus and its related combination viruses. Summarized the weight changes, lung viral titers and fatality rate of each group GMS virus for comprehensive analysis. a: 54-56; b: 87-89; c: 127-129; d: 148-150; e: 178-180; f: 188-190; g: 200-202 and 295-297; h: 265-267; i: 267-269; j: 280-282; WT and PBS in every group were colored with red and grey. In every group, different GMS viruses were represented with various colors according to the pathogenicity to mice. Among them, dark blue represented the strongest pathogenicity in this group, followed by purple, next orange and green, and finally light pink, indicating the weakest pathogenicity to mice in this group.
